# Targeting neurons in the gastrointestinal tract to treat Parkinson's disease^☆^

**DOI:** 10.1016/j.prdoa.2019.06.001

**Published:** 2019-07-02

**Authors:** Robert A. Hauser, Dean Sutherland, Juan A. Madrid, Maria Angeles Rol, Steven Frucht, Stuart Isaacson, Fernando Pagan, Brian N. Maddux, George Li, Winona Tse, Benjamin L. Walter, Rajeev Kumar, Daniel Kremens, Mark F. Lew, Aaron Ellenbogen, Odinachi Oguh, Alberto Vasquez, William Kinney, Matt Lowery, Maria Resnick, Nicole Huff, Jerry Posner, Karla V. Ballman, Brian E. Harvey, Michael Camilleri, Michael Zasloff, Denise Barbut

**Affiliations:** a USF Parkinson's Disease & Movement Disorder Ctr., Tampa, FL, United States of America; b Sarasota Memory Hospital Clinical Research Ctr., Sarasota, FL, United States of America; c Chronobiology Laboratory, University of Murcia, CIBERFES, Spain; d New York University Langone Medical Ctr, New York, NY, United States of America; e Parkinson's Disease and Movement Disorder Ctr. of Boca Raton, Boca Raton, FL, United States of America; f Department of Neurology, Georgetown University Medical Ctr., Washington, DC, United States of America; g Riverhills Healthcare, Inc., Cincinnati, OH, United States of America; h MEDSOL Clinical Research, Port Charlotte, FL, United States of America; i Parkinson's and Movement Disorders Ctr., Icahn School of Medicine at Mount Sinai, New York, NY, United States of America; j Parkinson's & Movement Disorders Ctr., University Hospitals Cleveland Medical Center, Cleveland, OH, United States of America; k Rocky Mountain Movement Disorder Ctr., Englewood, CO, United States of America; l Department of Neurology, Thomas Jefferson University, Philadelphia, PA, United States of America; m Keck Hospital of USC, Los Angeles, CA, United States of America; n Quest Research Institute and Michigan Institute for Neurologic Disorders, Farmington, MI, United States of America; o Neuroscience Research, University of Florida, Jacksonville, FL, United States of America; p Suncoast Neuroscience Associates, Inc., St. Petersburg, FL, United States of America; q Enterin, Inc., Philadelphia, PA, United States of America; r Memorial Sloan-Kettering Cancer Ctr., NY, NY, United States of America; s Weill Cornell Medical Ctr., New York, NY; t Mayo Clinic, Rochester, MN, United States of America; u Medstar-Georgetown Transplant Institute, Washington, DC, United States of America

**Keywords:** Squalamine, ENT-01, Parkinson's disease, Constipation, Treatment, Non-motor, Synuclein

## Abstract

**Background:**

Parkinson's disease (PD) is associated with α-synuclein (αS) aggregation within the enteric nervous system (ENS) and constipation. Squalamine displaces proteins that are electrostatically bound to intracellular membranes and through this mechanism suppresses aggregation of αS monomers into neurotoxic oligomers.

**Objective:**

We sought to evaluate the safety of ENT-01 oral tablets (a synthetic squalamine salt), its pharmacokinetics, and its effect on bowel function in PD patients with constipation.

**Methods:**

In Stage 1, 10 patients received escalating single doses from 25 to 200 mg/day or maximum tolerated dose (MTD). In Stage 2, 34 patients received daily doses escalating from 75 to a maximum of 250 mg/day, a dose that induced change in bowel function or MTD, followed by a fixed dose for 7 days, and a 2-week washout. Primary efficacy endpoint was defined as an increase of 1 complete spontaneous bowel movement (CSBM)/week, or 3 CSBM/week over the baseline period, as defined by FDA guidelines for prokinetic agents. Safety was also assessed.

**Results:**

Over 80% of patients achieved the primary efficacy endpoint, with the mean number of CSBM/week increasing from 1.2 at baseline to 3.6 during fixed dosing (*p* = 1.2 × 10^−7^). Common adverse events included nausea in 21/44 (47%) and diarrhea in 18/44 (40%) patients. Systemic absorption was <0.3%.

**Conclusions:**

Orally administered ENT-01 was safe and significantly improved bowel function in PD, suggesting that the ENS is not irreversibly damaged in PD. Minimal systemic absorption suggests that improvements result from local stimulation of the ENS. A double-blind, placebo-controlled study is now ongoing.

## 1 Introduction

Parkinson's disease (PD) is a progressive neurodegenerative disorder caused by accumulation of the protein α-synuclein (αS) within the brain, autonomic nerves, and enteric nervous system (ENS) [1]. While motor symptoms are still required for a diagnosis of PD [2], non-motor symptoms often represent a greater therapeutic challenge [3]. These symptoms include constipation [4,5], disturbances in sleep architecture [6,7], cognitive dysfunction [8], hallucinations [9,10], REM behavior disorder (RBD) and depression [5,11], all of which result from impaired function of neural pathways not restored by dopamine replacement. In fact, long-term institutionalization, caregiver burden and decreased life expectancy correlate more significantly with severity of non-motor symptoms than with motor symptoms [12].

In PD, neurotoxic aggregates of αS accumulate in the ENS [13,14], as they do in the central nervous system (CNS). In addition, both epidemiological and preclinical studies suggest that aggregates of αS formed within the ENS might underlie the etiology of the CNS component of PD [14,15]. Examination of the ENS from PD patients suggests that gastrointestinal dysmotility is not a consequence of neuronal loss, but more likely the result of dysfunction caused by the presence of aggregated αS within specific neurons [16].

We have focused on squalamine, an antimicrobial aminosterol discovered in the dogfish shark, *Squalus acanthias* [17]. Squalamine, a cationic lipid, is able to enter eukaryotic cells, including primary neurons [18], and displace proteins that are bound electrostatically to intracellular membranes with a strong negative surface charge [[18], [19], [20], [21]]. We recently reported that squalamine can effectively displace αS from membranes to which it is bound by this electrostatic mechanism [22]. In addition, we have shown *in vitro,* in cell culture, and in a *C. elegans* model of PD that through the mechanism of membrane displacement, squalamine also suppresses the primary nucleation and subsequent aggregation of αS monomers into neurotoxic oligomers [22].

Based on these and prior preclinical studies we conducted a 50-subject proof of principle Phase 2a clinical trial evaluating the oral administration of a synthetic salt of squalamine (ENT-01) in the treatment of severe constipation in patients with PD.

## 2 Materials and methods

### 2.1 Study design

This multicenter Phase 2 trial was conducted in two Stages: a dose-escalation toxicity study in Stage I and a dose range-seeking and proof of efficacy study in Stage 2. The protocol was reviewed and approved by the institutional review board for each participating center and patients provided written informed consent.

Following successful screening, all subjects underwent a 14-day run-in period where the degree of constipation was assessed through a validated daily log [23] establishing baseline complete spontaneous bowel movements (CSBM)/week. Subjects with an average of <3 CSBM/week proceeded to dosing.

In Stage 1, 10 PD patients received a single escalating dose of ENT-01 every 3–7 days beginning at 25 mg and continuing up to 200 mg or the limit of tolerability, followed by 2-weeks of wash-out. Tolerability limits included 4–6 stools/day over baseline and 3–5 episodes of vomiting over 24 h. A given dose was considered efficacious in stimulating bowel function (prokinetic) if the patient had a CSBM within 24 h of dosing.

**Fig. 1:**
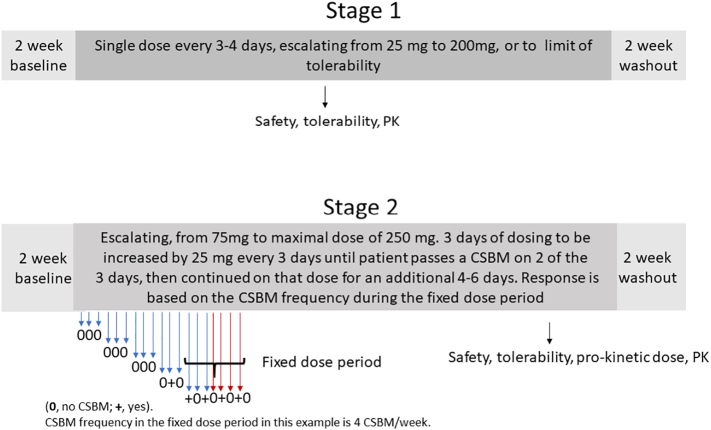
Phase 2a trial.

In Stage 2, 34 patients were evaluated. First, 14 new PD patients were administered ENT-01 daily, beginning at 75 mg, escalating every 3 days by 25 mg to a dose that had a clear prokinetic effect (CSBM within 24 h of dosing on at least 2 of 3 days at a given dose), or the maximum dose of 250 mg or the tolerability limit. This dose was then maintained (“fixed dose”) for an additional 3–5 days, amounting to at least 7 days on the fixed dose. This was then followed by a 2-week washout (Fig. 1).

### 2.2 Participants

Patients were between 18 and 86 years of age and diagnosed with PD by a clinician trained in movement disorders following the UK Parkinson's Disease Society Brain Bank criteria [24]. Patients had to have a history of constipation as defined by <3 CSBM/week and satisfy the Rome IV criteria for functional constipation [25] at screening, which requires 2 or more of the following: straining during at least 25% of defecations; lumpy or hard stools in at least 25% of defecations; sensation of incomplete evacuation in at least 25% of defecations; sensation of anorectal obstruction/blockage in at least 25% of defecations; manual maneuvers to facilitate at least 25% of defecations.

### 2.3 Study endpoints

The primary objectives were to evaluate the safety of orally administered ENT-01, proof of efficacy and the dose range required to achieve a bowel response. In Stage 2, a “responder” was defined by a CSBM increase of ≥1/week over baseline or ≥3CSBM/week during the “fixed-dose” period. Other constipation related parameters monitored included spontaneous bowel movements (SBMs)/week, stool consistency (Bristol Stool Form Scale) [26,27], ease of passage (Ease of Evacuation Scale) [28], rescue medication use and symptoms and quality of life related to bowel function (PAC-SYM [29] and PAC-QOL [30]).

### 2.4 Neurologic symptoms

PD symptoms were assessed using the Unified Parkinson's Disease Rating Scale (UPDRS) [24]. Exploratory end-points included depression assessed using the Beck Depression Inventory (BDI-II) [31], cognition assessed using the Mini Mental State Examination (MMSE) [32], sleep and RBD using a daily diary and an RBD questionnaire (RBDQ) [33] and hallucinations assessed using the PD hallucinations questionnaire (PDHQ) [34] and direct questioning. Assessments were made at baseline and at the end of the fixed dose and washout periods.

### 2.5 Pharmacokinetics

PK data were collected on the 10 patients enrolled in Stage 1 and 10 patients enrolled in Stage 2 to determine the extent of systemic absorption. Squalamine ion concentrations were measured using a modification of a published LC/MS method [35].

### 2.6 Circadian system assessment

Circadian system functionality was evaluated by continuously monitoring wrist skin temperature using a temperature sensor (Thermochron iButton DS1922L; Maxim, Dallas) according to the procedure described in detail by Sarabia et al. in 2008 [36]. A nonparametric analysis was performed for each participant to characterize distal skin temperature (DST) as previously described [36,37]. Briefly, this analysis includes the following parameters: the inter-daily stability (the constancy of 24-hour rhythmic pattern over days, IS); intra-daily variability (rhythm fragmentation, IV); average of 10-minute intervals for the 10 h with the minimum temperature (L10); average of 10-minute intervals for the 5 h with the maximum temperature (M5) and the relative amplitude (RA), which was determined by the difference between M5 and L10, divided by the sum of both. Finally, the Circadian Function Index (CFI) was calculated by integrating IS, IV, and RA. Consequently, CFI is a global measure that oscillates between 0 for the absence of circadian rhythmicity and 1 for a robust circadian rhythm [37].

### 2.7 Statistical analysis

No formal size calculation was performed for Stage 1. The number of subjects (*n* = 10) was based on feasibility and was considered sufficient to meet the objectives of the study which was to determine the tolerability of the treatment across the range of tested doses. For Stage 2, assuming the highest proportion of spontaneous resolution of constipation with no treatment to be 0.10, 34 evaluable subjects with assessments at baseline and at the end of the fixed dose period provided 80% power to detect the difference between 0.10 (proportion expected if patients are not treated) and an ENT-01 treated proportion of 0.29.

Adverse events (AEs) were coded using the current version of MedDRA. Severity of AEs was assessed by investigators according to Common Terminology Criteria for Adverse Events (CTCAE, v4.03): Grade 1 is labeled as Mild, Grade 2 as Moderate, and Grade 3 and above as Severe. AEs that have a possible, probable or definite relationship to study drug were defined to be related to the study drug while others were defined as “not related”. The numbers (percentage) of subjects who experienced an AE during escalation and fixed dosing periods were summarized for each stage. The denominator for calculating the percentages were based on the number of subjects ever exposed overall.

The primary efficacy outcome variable was whether or not a subject was a “success” or “failure”. This is an endpoint based on subject diary entries for the “fixed dose” period prior to the endpoint assessment defined as an average complete stool frequency increase by 1 or more over baseline, OR 3 or more complete spontaneous stools/week. The subject is deemed a “success” if s/he meets one or more of the criteria listed above, otherwise the subject will be deemed a “failure”. The primary analysis was based on all subjects with a baseline assessment and an assessment at the end of the “fixed-dose” period and was a comparison of the proportion of successes with 0.10 (the null hypothesis corresponding to no treatment effect). The proportion of subjects for whom the drug was a success was estimated with a binomial point estimate and corresponding 95% confidence interval.

An exploratory analysis was done with respect to sleep, body temperature, mood, fatigue, hallucinations, cognition and other motor and non-motor symptoms of PD. Continuous measurements within a subject were compared with a paired *t*-test and continuous measurements between subject groups were compared with a two-group *t*-test. Categorical data were compared with a chi-squared test or a Fisher's exact test if the expected cell counts were too small for a chi-squared test.

## 3 Results

Fifty patients were enrolled and 44 were dosed. Baseline characteristics of patients are shown in Table S1. There were equal proportions of males and females in Stage 1 as well as some non-Caucasian patients, but white males predominated in Stage 2. Patients in Stage 2 had somewhat longer duration of Parkinson's disease and higher UPDRS scores than participants in Stage 1.

### 3.1 Safety and adverse event profile

In Stage 1, 10 patients were dosed, 1 (10%) withdrew prior to completion and 9 (90%) completed dosing. In stage 2, 6 (15%) patients had ≥3 CSBM/week at the end of the run-in period and were excluded, 34 patients were dosed, and bowel response was assessable in 31 (91%). Two patients (5.8%) were terminated prior to completion because of recurrent dizziness, and 3 others withdrew during dosing (8.8%): 2 because of loose stools and 1 because of holiday. Study-drug assignments and patient disposition are shown in Table S2 and Fig. S1, respectively.

Most AEs were confined to the GI tract (88% in Stage 1 and 63% in Stage 2). The most common AE was nausea which occurred in 4/10 (40%) patients in Stage 1 and in 15/34 (50%) in Stage 2 (Table 1) and did not appear to be dose related. Loose stools, although classified as an AE, are an anticipated pharmacological response of ENT-01, and occurred in 4/10 (40%) patients in Stage 1 and 15/34 (44%) in Stage 2. Other GI-related AEs included abdominal pain 11/44 (32%), flatulence 3/44 (6.8%), vomiting 3/44 (6.8%), worsening of acid reflux 2/44 (4.5%), and worsening of hemorrhoids 1/44 (2.2%). One patient had a lower GI bleed (serious adverse event, SAE) during the withdrawal period. This patient was receiving aspirin, naproxen and clopidogrel at the time of the bleed, and colonoscopy revealed large areas of diverticulosis and polyps. This SAE was considered unrelated to study medication.

**Table 1:** All adverse events (*n*, %).

Enrolled	Stage 1 (*n* = 10)	Stage 2 (*n* = 40)
Dosed	10	34
Gastrointestinal		
Nausea		
Mild	4 (40)	16 (47)
Moderate	0	1 (2.9)
Diarrhea		
• Mild	1 (10)	12 (35)
• Moderate	3 (30)	2 (5.8)
• Severe	0	1 (2.9)
Vomiting		
• Mild	1 (10)	2 (5.8)
• Moderate	0	0
Abdominal pain		
• Mild	2 (20)	4 (11.7)
• Moderate	3 (30)	2 (5.8)
Flatulence		
• Mild	2 (20)	1 (3)
• Moderate	0	0
Loss of appetite^a^		
• Mild	1 (10)	0
• Moderate	0	0
Worsening acid reflux		
• Mild	0	2 (5.8)
• Moderate	0	0
Worsening hemorrhoid		
• Mild	0	1 (3)
• Moderate	0	0
Lower GI bleed^b^		
• Severe	0	1 (2.5)
Non-gastrointestinal		
Dizziness		
• Mild	0	7 (20.5)
• Moderate	0	1 (2.9)
Blood in urine^a^		
• Mild	1 (10)	0
• Moderate	0	0
Headache		
• Mild	1 (10)	3 (8.8)
• Moderate	0	0
Urinary retention		
• Mild	0	1 (3)
• Moderate	0	0
Urinary tract infection		
• Mild	0	1 (3)
• Moderate	0	2 (5.8)
Increased urinary frequency		
• Mild	0	2 (5.8)
• Moderate	0	0
Skin lesions-rash		
• Mild	0	3 (8.8)
• Moderate	0	0
Eye infection		
• Mild	0	1 (3)
• Moderate	0	0
Difficulty falling asleep		
• Mild	0	1 (3)
• Moderate	0	0

a Unrelated to ENT-01.

b Colonic diverticulosis, polyp, patient on aspirin, Plavix and naproxen. Unrelated to ENT-01.

The only other noteworthy AE was dizziness 8/44 (18%). Dizziness was graded as moderate in one patient who was receiving an alpha-adrenergic blocking agent (Terazosin). This patient was withdrawn from the study and recovered spontaneously. All other AEs resolved spontaneously without discontinuation of ENT-01. The relationship between dose and AEs is shown in Table S3. Dose limiting toxicity criteria are presented in Table S4.

### 3.2 Effects on bowel function

**Fig. 2:**
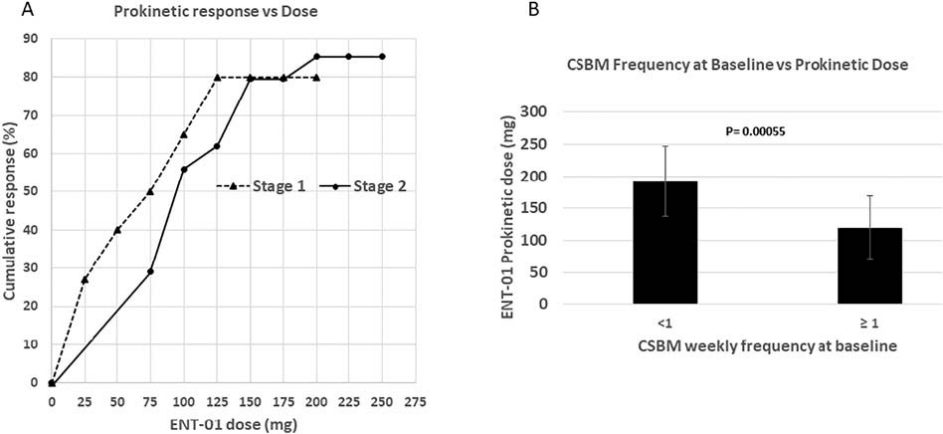
Prokinetic activity of ENT-01. A. In Stage 1 (single dose), cumulative prokinetic response rate was defined as the proportion of patients who had a CSBM within 24 h of dosing. Dosing started at 25 mg. In Stage 2 (daily dosing), a prokinetic response was defined as the fraction of patients who had a CSBM within 24 h of dosing on at least 2 out of 3 days at any given dose. Dosing started at 75 mg. B. Prokinetic dose of ENT-01 was significantly related to baseline constipation severity (*p* = 0.00055). Patients with baseline CSBM <1 required a higher dose (mean, 192 mg) of ENT-01 than patients with CSBM ≥1 (mean, 120 mg). Significance determined by *t*-test.

Cumulative responder rates for bowel function are shown in Fig. 2A. In Stage 1 (single dose), cumulative response rate increased in a dose-dependent fashion from 25% at 25 mg to a maximum of 80% at 200 mg.

In Stage 2 (daily dosing), the response rate increased in a dose-dependent fashion from 29.4% at 75 mg to 82.4% at 250 mg. The dose required for a bowel response was patient-specific and varied from 75 mg to 250 mg. Median efficacious dose was 100 mg. During the dosing titration, patients rarely experienced a CSBM until their prokinetic dose had been reached, after which the frequency sharply increased.

Average CSBM/week increased from 1.2 at baseline to 3.6 at fixed dose (*p* = 1.2 × 10^−7^) and SBM increased from 2.6 at baseline to 4.4 at fixed dose (*p* = 1.2 × 10^−5^) (Table 2). Use of rescue medication decreased from 1.8/week at baseline to 0.3/week at fixed dose (*p* = 2.33 × 10^−5^). Stool consistency based on the Bristol stool scale also improved, increasing from (a mean of) 2.7 to 4.1 (*p* = 0.0002) and ease of passage increased from 3.2 to 3.7 (*p* = 0.04). Subjective indices of wellbeing (PAC-QOL) and constipation symptoms (PAC-SYM) also improved during treatment (*p* = 0.01 and *p* = 0.05 respectively).

**Table 2:** Stool related indices in Stage 2 (Dosed patients, *n* = 34).

	Baseline (mean, SD)	Fixed dose (mean, SD)	*p*-Value
CSBM^a^	1.2 (0.90)	3.6 (2.35)	1.2 × 10^−7^
SBM^a^	2.6 (1.45)	4.4 (2.16)	1.2 × 10^−5^
Suppository use^a^	1.8 (1.92)	0.3 (0.67)	2.3 × 10^−5^
Consistency^c^	2.7 (1.20)	4.1 (2.15)	0.0002
Ease of passage^b^	3.2 (0.73)	3.7 (1.19)	0.04
PAC-QOL total	1.4 (0.49)	1.2 (0.59)	0.01
PAC-SYM	1.3 (0.45)	1.1 (0.49)	0.05

a Weekly average.

b Ease of evacuation scale, where 1-manual disimpaction and 7 = incontinent.

c Bristol stool scale 1–7, where 1 = separate hard lumps and 7 = liquid consistency.

The dose that proved efficacious in inducing a bowel response was strongly related to constipation severity at baseline (*p* = 0.00055) (Fig. 2B, lower panel); patients with baseline constipation of <1 CSBM/week required higher doses for a response (mean 192 mg) than patients with ≥1 CSBM/week (mean 120 mg).

The improvement in most stool-related indices did not persist beyond the treatment period, returning to baseline value during the washout period (Table S5).

### 3.3 Effect of ENT-01 on neurologic symptoms in PD

During the trial, neuropsychiatric symptoms were assessed in the 34 patients who were dosed in Stage 2 at baseline and at the end of the fixed dose and washout periods (Table S6). Total UPDRS score was 64.2 at baseline, 60.8 at the end of the fixed dose period, and 55.7 at the end of the 2-week wash-out period (*p* = 0.0008); MMSE was 28.4 at baseline, 28.8 at the end of the fixed dose period, and 29.3 at the end of wash-out (*p* = 0.0004). PDHQ was 1.3 at baseline and 0.9 during wash-out (*p* = 0.03). Hallucinations and delusions improved or disappeared altogether in 5 of 6 patients during treatment and did not return for several weeks following discontinuation of ENT-01. The frequency of arm or leg thrashing during sleep (RBD) diminished progressively from 2.2 episodes/week at baseline to 0 at maximal dose and total sleep time increased from 7.1 h at baseline to 8.4 h at 250 mg (Fig. S2).

### 3.4 ENT-01 and circadian rhythm

We evaluated wrist skin temperature by continuous monitoring (Thermochron iButton DS1922L; Maxim, Dallas) following published procedures (36). Circadian rhythm of skin temperature was evaluable in 12 patients. ENT-01 administration improved all markers of healthy circadian function, increasing rhythm stability (IS, *p* = 0.026), relative amplitude (RA, *p* = 0.001) and circadian function index (CFI, *p* = 0.016), while reducing rhythm fragmentation (IV, *p* = 0.031). The improvement persisted during wash-out (IS, *p* = 0.008 and CFI, *p* = 0.004) (Fig. S3). These data indicate that orally administered ENT-01 appears to improve circadian rhythm.

### 3.5 ENT-01 acts locally on the ENS

To determine the extent of systemic absorption, we collected blood at pre-specified times as indicated in Methods (Table S7). Systemic absorption of ENT-01 was <0.3%, strongly suggesting that its effect is mediated by local action in the GI tract.

## 4 Discussion

We demonstrate in this study that squalamine, the active ion of ENT-01, can restore gastrointestinal motility in patients with PD. This suggests that a major division of the nervous system, the ENS, is not irreversibly damaged in patients with PD, despite the often-long-standing constipation that might suggest otherwise. From the limited studies conducted on human tissue, it is believed that constipation is caused by the accumulation of aggregates of αS, rather than by loss of neurons, but the underlying mechanism is unknown [16]. In PD mouse models neurological dysfunction has been shown to correlate with the accumulation of aggregates of A53T αS within the affected neurons and their axonal synapses [38,39].

Although the mechanism by which these aggregates disturb neuronal function is still unclear in these mouse models, it is assumed that because they are membrane active [22], αS aggregates interfere with cellular functions. In addition, recent studies support the hypothesis that the aggregation of αS monomers is initiated on a membrane surface enriched in anionic phospholipids [22]. For these reasons, we considered targeting the ENS in patients with PD with orally administered ENT-01, a compound known to displace proteins that are bound electrostatically to intracellular membranes. The squalamine ion rapidly activates the AMPA glutamate receptor in primary cortical neurons by displacing the inhibitory TARP regulatory protein from the cytoplasmic face of the plasma membrane [18]; similarly the squalamine ion inhibits the sodium hydrogen exchanger (type 3) by displacing its positively charged carboxyl-terminus from the cytoplasmic face of the plasma membrane [21]. Most importantly, we have shown that the squalamine ion can displace αS monomers from anionic membranes and inhibit its aggregation into neurotoxic aggregates *in vitro* and *in vivo* [22].

This phase 2 trial involving 50 patients with PD and constipation assessed the safety of orally administered ENT-01, and the effect on bowel function and neurologic symptoms of PD. In addition, the study aimed to identify a dose of ENT-01 that normalized bowel function in each patient. While the study achieved the objectives of identifying safety and pharmacodynamic responses supporting further evaluation of ENT-01 in PD, it is the first proof-of-concept demonstration that pharmacologically targeting the affinity of neuronal membranes for αS can restore GI motility in patients with PD and constipation. ENT-01 most likely improves bowel function in PD patients by acting locally on the gastrointestinal tract as supported by the oral bioavailability <0.3%. This conclusion is further supported by prior clinical trials that demonstrated that intravenous administration of squalamine was not associated with increased gastrointestinal motility, despite reaching systemic blood levels one thousand-fold greater than that achieved by orally administered ENT-01 [40,41]. The topical action would also explain why AEs were largely confined to the gastrointestinal tract.

Although ENT-01 stimulates GI motility in PD patients with constipation, we have no direct experimental evidence that it does so by the proposed mechanism of electrostatic displacement of αS aggregates from ENS neurons. Conclusive electrophysiological studies will be difficult to conduct on human tissues. Our hypothesis, however, also predicts that oral administration of ENT-01 should, over the course of chronic administration, reduce the accumulation of neurotoxic αS aggregates within the ENS of a PD patient. These studies will require endoscopic sampling of upper GI tissues taken before and during ENT-01 administration. The feasibility of such a study is being assessed.

The principal AEs were nausea and loose stools. Nausea was not dose related and possibly positional since it occurred most frequently when taken supine. Loose stools are an expected pharmacological response at high doses and are reversed by reducing the dose.

The effective pro-kinetic dose ranged between 75 mg and 250 mg, with 82.4% of patients responding within this range. This dose correlated positively with constipation severity at baseline, consistent with the hypothesis that gastrointestinal dysmotility in PD results from the progressive accumulation of αS in the ENS, and that the effective intracellular concentrations of squalamine required to restore neuronal activity would be proportional to the intracellular load of αS. Confirmation of this hypothesis would require examination of gastrointestinal tissue from the clinical population.

We observed potential signals of clinical benefit for parkinsonism, cognition, hallucinations, sleep and RBD. Although intriguing, this was an open label trial and placebo effects cannot be excluded; these outcomes must undergo rigorous evaluation in future placebo-controlled trials.

We also monitored circadian rhythm through the use of a temperature sensor that continuously captured wrist skin temperature [36], an objective measure of the autonomic regulation of vascular perfusion. Circadian cycles of wrist skin temperature have been shown to correlate with sleep-wake cycles, reflecting the impact of nocturnal heat dissipation from the skin on the decrease in core temperature and the onset of sleep [36,37]. Oral administration of ENT-01 had a significant positive impact on the circadian rhythm of skin temperature in the 12 patients with evaluable data. Precisely how ENT-01 influenced circadian rhythm remains under investigation and will be pursued further in additional clinical studies.

In summary, orally administered squalamine stimulates GI motility in both mouse models of PD and patients with PD and constipation. Placebo controlled clinical trials of longer duration are planned to determine the durability of the effect of oral ENT-01 on constipation in PD and possible effects on neurologic symptoms and disease progression.

## Declaration of Competing Interest

Barbut, Zasloff, Kinney, Harvey, Resnick, Lowry, and Huff are employees of Enterin and hold equity. Camilleri is a member of Enterin's Scientific Advisory Board. Ballmann is a paid consultant of Enterin. Hauser has received consulting fees from Enterin.

## Appendix A Supplementary data

Supplementary material Image 1
