# Supplementary material for: Targeting neurons in the gastrointestinal tract to treat Parkinson's disease
Source: Clin Park Relat Disord. 2019 Jul 2;1:2–7. doi: 10.1016/j.prdoa.2019.06.001 (PMC8288812; doi:10.1016/j.prdoa.2019.06.001)
Supplement: Supplementary file 1 — Supplementary material [file mmc1.docx]

**Supplementary Information**

**Targeting Neurons in the Gastrointestinal Tract to Treat Parkinson’s Disease**

**Table of contents:**

- List of investigators
- Figure S1: Patient disposition
- Figure S2: Effect of ENT-01 on total sleep time
- Figure S3: Effect of ENT-01 on circadian rhythm parameters
- Table S1: Baseline characteristics of dosed patients
- Table S2: Study drug assignments and adherence to treatment
- Table S3: Common Adverse events by dose
- Table S4: Dose limiting toxicity criteria
- Table S5: Reversal of stool indices to baseline during wash-out (Stage 2)
- Table S6: Effect of ENT-01 on neurological symptoms
- Table S7: PK of orally administered ENT-01 in Stages 1 and 2
- Financial disclosures
- References

**List of investigators:**

Robert A. Hauser, M.D., M.B.A., USF Parkinson’s Disease & Movement Disorder Ctr. Tampa, Florida

Juan A. Madrid, Chronobiology Laboratory, IMIB-Arrixaca, Universidad de Murcia, CIBERFES, Instituto de Salud Carlos III, Murcia, Spain

Angeles Rol, Chronobiology Laboratory, IMIB-Arrixaca, Universidad de Murcia, CIBERFES, Instituto de Salud Carlos III, Murcia, Spain

Dean Sutherland, M.D., Ph.D., Sarasota Memory Hospital Clinical Research Ctr., Sarasota, Florida

Stuart Isaacson, M.D., Parkinson’s Disease and Movement Disorder Ctr. Of Boca Raton. Boca Raton, Florida

Fernando Pagan, M.D., Georgetown University, Department of Neurology, Washington, D.C.

Brian N. Maddux, M.D., Ph.D., Riverhills Healthcare, Inc. Cincinatti, Ohio

George Li, M.D., MEDSOL Clinical Research, Port Charlotte, Florida

Winona Tse, M.D., Icahn School of Medicine at Mount Sinai, Parkinson's and Movement Disorders Center, New York, NY.

Benjamin L. Walter, M.D., Parkinson’s & Movement Disorders Center, University Hospitals Cleveland Medical Center, Cleveland, Ohio

Rajeev Kumar, M.D., Rocky Mtn. Movement Disorder Ctr., Englewood, Colorado.

Daniel Kremens, M.D., Thomas Jefferson University, Department of Neurology, Philadelphia, PA

Mark F. Lew, M.D., Keck Hospital of USC, Los Angeles, CA

Aaron Ellenbogen, D.O., Quest Research Institute and Michigan Institute for Neurologic Disorders, Farmington, Michigan

Odinachi Oguh, M.D., Neuroscience Research, University of Florida, Jacksonville, Florida

Alberto Vasquez, M.D., Suncoast Neuroscience Associates, Inc. St. Petersburg, Florida

**FIGURE S1**

**FIGURE S1 Patient disposition in Stage 2**

Patients enrolled (n=40)

Failed to meet dosing criteria (n=6)

Dosed (n=34)

Discontinued (n=5)

- Withdrew consent (n=3)^1^
- Adverse event (n=2)^2^

Prokinetic response assessable (n=31)

Completed dosing (n=29)

^1^ 1 patient was lost to follow up and 2 patients discontinued because of diarrhea

^2^ 2 patients were withdrawn because of recurrent dizziness after medication

**Figure S2**

**Figure S2. Total sleep time in relation to ENT-01 dose.**

Total sleep time was obtained from the sleep diary by subtracting awake time during the night from total time spent in bed. Total sleep time per night was logged for each patient at baseline, each dosing period and at washout and the means were determined. The light grey bar represents the baseline value for each cohort at a given dose level and the dark grey bar represents the value for the same cohort at the stated dose of ENT-01. The number of patients represented at each value are: Baseline,33;75mg,21;100mg,28;125mg,18;150mg,15;175mg,12;200mg,7;225mg,3; 250mg,2; washout, 33. P values were as follows: 75mg, p=0.4; 100mg, p=0.1; 125mg, p=0.3; 150mg, p=0.07; 175mg, p=0.03; 200mg, p=0.3; 225mg, p=0.5; 250mg, p=0.3; wash-out, p=0.04 (paired t test).

**Figure S3**

**A**

**
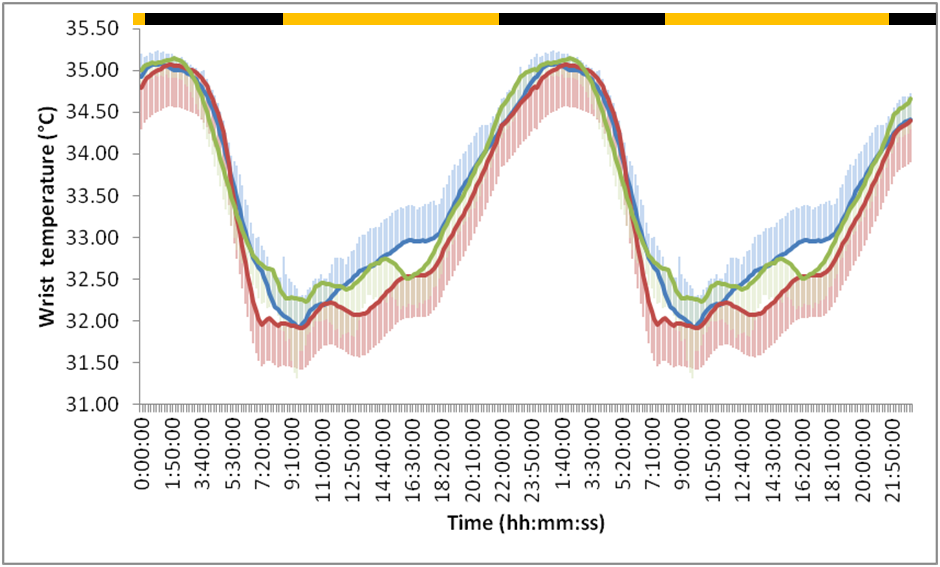
**

**B**


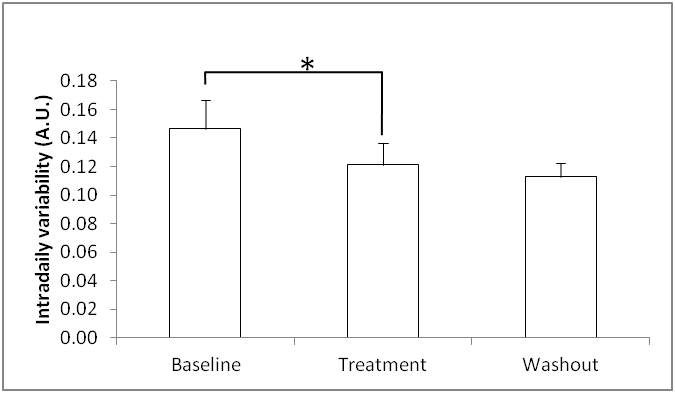

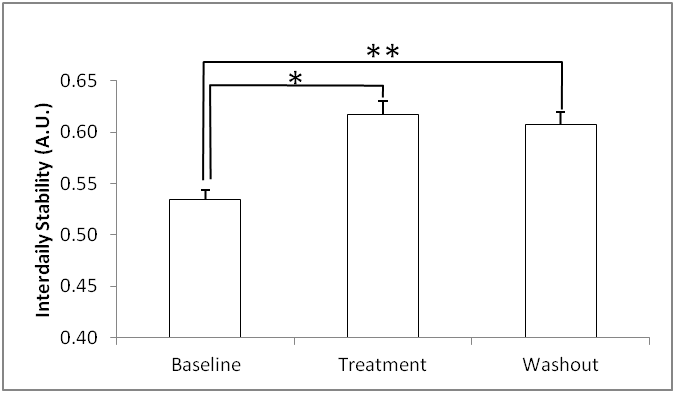


**
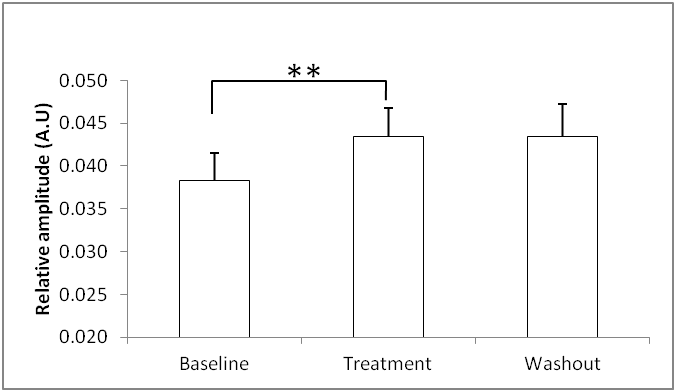

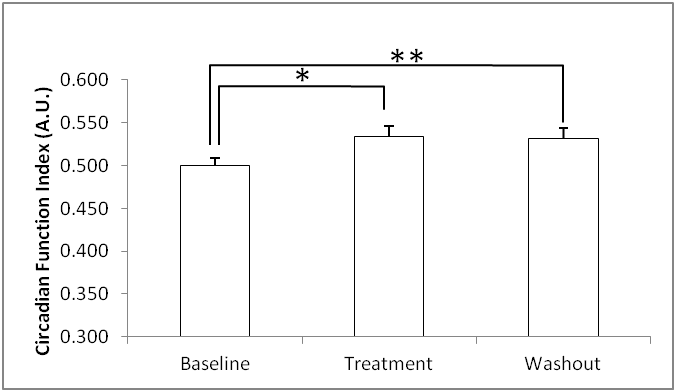
**

**
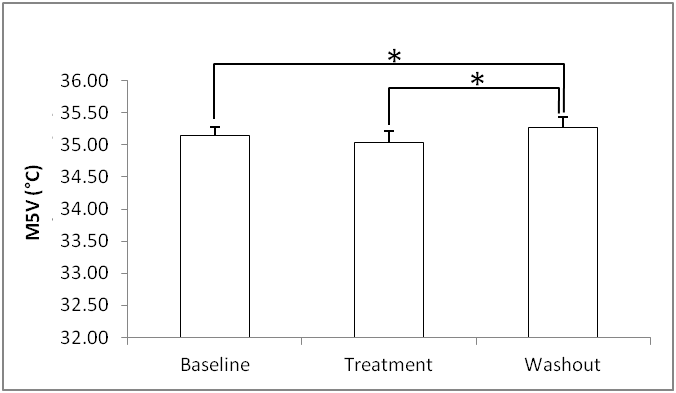

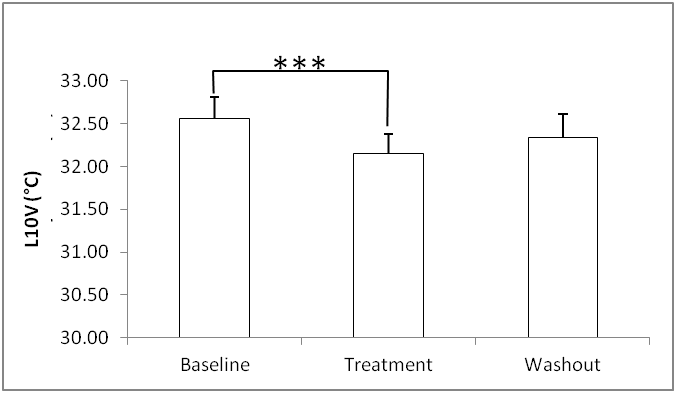
**

**Figure S3.** **Effect of ENT-01 on circadian rhythm**. A) Mean waveform of temperature under three conditions per patient: baseline (blue line), treatment with highest drug dose (red line) and washout (green line). Each mean waveform is double plotted for better visualization. Low temperatures indicates higher activation, while higher values are associated with drowsiness and sleepiness. The top black bar indicates a standard rest period from 23:00 to 07:00h.

B) Circadian non- parametric analysis of wrist skin temperature rhythm throughout each condition (baseline, treatment with highest dose of ENT-01 and washout). Inter-daily stability (IS) or rhythm regularity, intra-daily variability (IV), or rhythm fragmentation, relative amplitude (RA) or rhythm’s robustness. M5V refers to the five consecutive hours with the highest temperature or high somnolence, while L10V, indicates the mean of the ten consecutive hours with lowest temperature or high activation. The circadian function index (CFI) is an integrated score that ranges from 0 (absence of circadian rhythm) to 1 (robust circadian rhythm). Student’s paired t-test, *p < .05, **p < 01, ***p<.001.Values expressed as mean ± SEM (n=12 in each condition).

| **Table S1. Baseline Characteristics of Dosed Patients** | | | |
| --- | --- | --- | --- |
| **Characteristic** | **Stage 1** (n=10)** | **Stage 2*** (n=34)** | **Total (n=44)** |
| **Sex- no. (%)** | | | |
| 1.          Male | 5 (50) | 25 (73.5) | 30 (68.1) |
| 1.          Female | 5 (50) | 9 (26.5) | 14 (31.8) |
| **White race-no. (%)** | 8 (80) | 34 (100) | 42 (95.5~~4~~) |
| **Age-year** | | | |
| Mean | 65.0 | 74.5 | 72.5 |
| Range | 58-70.5 | 60.6-84.2 | 58 – 84.2 |
| **Age at PD diagnosis-year** | | | |
| Mean | 61.1 | 67.7 | 66.2 |
| Range | 54.2-69 | 50.6-82.5 | 50.6 – 82.5 |
| **Duration of PD-year** | | | |
| Mean | 4.2 | 6.8 | 6.2 |
| Range | 1—11 | 0.3-17.3 | 0.3 – 17.3 |
| **Duration of constipation-year** | | | |
| Mean | 25.8 | 16.8 | 18.9 |
| Range | 1-65 | 0.5-66.0 | 0.5 – 66.0 |
| **UPDRS score** |  |  |  |
| Mean | 53.4 | 63.2 | 61.3 |
| Range | 33-88 | 24-122 | 24.0 – 122.0 |
| **Hoehn and Yahr-Stage** |  |  |  |
| Mean | 2.0 | 2.4 | 2.3 |
| Range | 2.0 | 1.0-5.0 | 1.0 – 5.0 |
| **Constipation severity* – CSBM/week- no. (%)** |  |  |  |
| 0-1 | 8(80) | 14(41.2) | 22 (50) |
| 1.1-2 | 2 (20) | 17(50) | 19 (43.2) |
| 2.1-3 | 0 | 3 (8.8) | 3 (6.8) |

*At baseline. Baseline value is the average number of CSBMs per week calculated at the end of the 2-week run-in period.

**In Stage 1, 10 patients received single escalating doses every 3-7 days starting at 25mg and escalating up to dose limiting toxicity (DLT) or 200mg, whichever came first, followed by a 2-week wash-out period.

***In Stage 2, 15 patients received daily doses starting at 75mg and escalating every 3 days up to prokinetic dose (dose producing CSBMs on at least 2 of 3 days) or 175mg, whichever came first, followed by an additional 2-4 days at that dose (“fixed dose” period). Wash-out lasted 2 weeks. The remaining 19 patients were escalated from 100mg to prokinetic dose or 250mg, whichever came first, followed by an additional 2-4 days at that dose and then a 2-week wash-out period.

| **Table S2. Study drug assignments and adherence to treatment** | | |
| --- | --- | --- |
|  | **Stage 1** | **Stage 2** |
| Enrolled | 10 | 40 |
| Failed prior to dosing | 0 | 6 |
| Dosed | 10 | 34 |
| - 25-200 | 10 |  |
| - 75-175 |  | 19 |
| - 100-250 |  | 15 |
| Terminated (%) | 0 (0) | 2 (5.8) |
| Withdrew (%) | 1 (10) | 3 (8.8) |
| Completed dosing (%) | 9 (90) | 31* (91) |
| Randomized |  | 15 |
| - Treatment |  | 6 |
| - Placebo |  | 9 |
| *29 patients completed dosing but an additional 2 who withdrew had an assessable prokinetic end-point. | | |

| **Table S3. Common adverse events by dose** | | | | | | |
| --- | --- | --- | --- | --- | --- | --- |
|  |  | | |  | | |
|  | Stage 1 | | | Stage 2 | | |
| Dose (mg) | Diarrhea | Nausea | Vomiting | Diarrhea | Nausea | Dizziness* |
| 0 | 0 | 0 | 0 | 1 | 0 | 2 |
| 25 | 1 | 0 | 0 | - | - | - |
| 50 | 1 | 0 | 0 | - | - | - |
| 75 | 1 | 0 | 0 | 7 | 3 | 8 |
| 100 | 0 | 1 | 1 | 10 | 12 | 7 |
| 125 | 1 | 2 | 1 | 3 | 4 | 8 |
| 150 | 1 | 0 | 0 | 2 | 11 | 2 |
| 175 | 1 | 1 | 0 | 1 | 12 | 0 |
| 200 | 0 | 2 | 0 | 3 | 6 | - |
| 225 | - | - | - | 3 | 1 |  |
| 250 | - | - | - | 2 | - |  |
| *lightheadedness included | | | | | | |

| **Table S4. Dose limiting toxicity criteria** | |
| --- | --- |
| **Diarrhea** | Increase 4-6 stools /day over baseline |
| **Vomiting** | 3-5 episodes in 24 hours |
| **Abdominal pain** | Moderate pain limiting daily activities |
| **Postural hypotension** | Moderately symptomatic and limiting daily activities or BP <80/40 |

| **Table S5. Reversal of stool indices to baseline during the wash-out period (Stage 2)** | | | | |
| --- | --- | --- | --- | --- |
|  | Baseline  (Mean, SD) | Fixed dose  (Mean, SD) | Wash-out  (Mean, SD) | P-value  (wash-out vs. baseline) |

| CSBM | 1.2 (0.90) | 3.6 (2.35) | 1.8 (1.19) | 0.01 |
| --- | --- | --- | --- | --- |
| SBM | 2.6 (1.45) | 4.4 (2.16) | 3.2 (1.80) | 0.16 |
| Ease | 3.2 (0.73) | 3.7 (1.19) | 3.3 (0.81) | 0.78 |
| Consistency | 2.7 (1.20) | 4.1 (2.15) | 2.8 (1.39) | 0.85 |
| Rescue meds | 1.8 (1.92) | 0.3 (0.68) | 1.0 (1.40) | 0.10 |
| PAQ-QOL | 1.4 (0.49) | 1.2 (0.59 | 1.2 (0.62) | 0.05 |
| PAQ-SYM | 1.3 (0.45) | 1.1 (0.47) | 1.1 (0.50) | 0.09 |

| **Table S6. Effect of ENT-01 on neurological symptoms (n=34)** | | | | | |
| --- | --- | --- | --- | --- | --- |
| **UPDRS** | **Baseline**  (Mean, SD) | **Fixed dose**  (Mean, SD) | **P-value** | Wash-out  (Mean, SD) | **P-value** |
| - **Part 1 (NMS)** | 11.5 (6.49) | 10.6 (6.08)) | 0.33 | 9.5 (5.17) | 0.08 |
| - **Part 2 (Daily living)** | 14.9 (7.99) | 14.7 (8.88) | 0.77 | 14.1 (8.06) | 0.40 |
| - **Part 3 (Motor)** | 35.4 (14.13) | 33.6 (15.07) | 0.17 | 30.3 (12.75) | 0.006 |
| - **Total** | 64.2 (23.36) | 60.8 (25.20) | 0.12 | 55.4 (22.82) | 0.0008 |
| **MMSE** | 28.4 (1.72) | 28.8 (1.88) | 0.17 | 29.3 (1.05) | 0.0004 |
| **PDHQ** | 1.3 (2.95) | 1.7 (3.30) | 0.45 | 0.9 (2.29) | 0.03 |
| **BDI-II** | 10.9 (7.45) | 9.8 (6.84) | 0.12 | 8.6 (5.40) | 0.09 |
| UPDRS: Unified Parkinson’s Disease Severity Score; NMS: Non-motor symptoms; BDI: Beck Depression Index-II; MMSE: Mini-mental State exam. PDHQ: Parkinson’s Disease Hallucination Questionnaire | | | | | |

**Table S7. Pharmacokinetics of orally administered ENT-01 in Stage 1**

**Stage 1**

| Dose (mg) | # of patients | Cmax (ng/ml) | T max (hour)  (Median Value) | T_1/2_ (hours)  (n) | AUC _0-8hr_ (ng*hour/ml | AUC_0-16hr_  (ng*hour/ml |
| --- | --- | --- | --- | --- | --- | --- |
| 25 | 9 | 2.84 | 1.0 | 2.6 (3) | 10.8 | 19.6 |
| 50 | 10 | 3.73 | 2.0 | 3.4 (3) | 18.5 | 33.1 |
| 75 | 9 | 4.33 | 2.0 | 2.8 (2) | 18.4 | 29.8 |
| 100 | 9 | 6.18 | 2.0 | 3.9 (5) | 29.6 | 51.5 |
| 125 | 9 | 9.63 | 2.0 | 3.9 (4) | 43.1 | 77.7 |
| 150 | 7 | 6.27 | 2.0 | 5.6 (4) | 31.5 | 64.0 |
| 175 | 7 | 10.3 | 2.0 | 9.1 (6) | 49.7 | 91.2 |
| 200 | 6 | 15.1 | 2.0 | 9.0 (5) | 78.3 | 157 |

The mean Cmax, Tmax and T_1/2_ and AUC of the squalamine ion following ENT-01 oral dosing for Stage 1 patients. The PK analyses are only approximate, as the lower limit of the validated concentration range was 10 ng/ml; most of the measured concentrations fell below that value.

**ROLE OF FUNDING SOURCE:**

Enterin funded the conduct of this research and was responsible for the study design, data collection, and analysis with input from invetsigators/authors as noted. Enterin participated in the interpretation of data, in the writing of the report, and in the decision to submit the article for publication along with investigators/authors as noted.

**FINANCIAL DISCLOSURES:**

**Barbut, Zasloff, Kinney, Harvey, Resnick, Lowry, and Huff** are employees of Enterin and hold equity**. Camilleri** is a member of Enterin’s Scientific Advisory Board**. Ballmann** is a paid consultant of Enterin**. Hauser** has received consulting fees from Enterin.

**Robert Hauser:**

Dr. Robert Hauser reports consulting fees from: Enterin, AbbVie Inc., Acorda Therapeutics, Academy for Continued Healthcare Learning, Acadia Pharmaceuticals, Inc., Adamas Pharmaceuticals, AstraZeneca, ApoPharma, Back Bay Life Science, Biotie Therapies, Bracket, Cerecor, Inc., ClearView Healthcare Partners, ClinicalMind Medical and Therapeutic Communications, CNS Ratings, LLC., Cowen and Company, Cynapsus Therapeutics, DDB Health LLC, Decision Resources Group (DRG), Eli Lilly & Company, eResearch Technology, Inc., Expert Connect, Extera Partners, GE Healthcare, Health Advances, HealthLogix, Health and Wellness Partners, Huron Consulting Group, Impax Laboratories, Impel Neuropharma, Intec Pharma Ltd., Jazz Pharmaceuticals, Kashiv Pharma LLC, Kyowa Kirin Pharmaceutical Development, Ltd., LCN Consulting, LifeMax, Life Sciences, Lundbeck LLC, The Lockwood Group, MEDACorp, Medscape, Medtronic, Michael J. Fox Foundation, Mitsubishi Tanabe Pharmaceuticals, Movement Disorder Society, National Institutes of Health (NIH), Neurocea LLC, Neurocrine Biosciences, Neuroderm, Neuropore Therapies, Orbes Medical Group, Outcomes Insights, Parkinson Study Group, Peerview Press, Pennside Partners, Pfizer, Inc., Pharma Two B, Ltd, Phase Five Communications, Prescott Medical Group, Prexton Therapeutics, Prilenia Development Ltd., Projects in Knowledge, Putnam Associates, Quintiles, RMEI Medical Education for Better Outcomes, SAI Med Partners LLC, Sarepta Therapeutics, Schlesinger Associates, Scion Neurostim, LLC, Seagrove Partners, LLC, Seelos, Slingshot Insights, Sunovion Pharmaceuticals, Inc., Sun Pharma, Teva Pharmaceutical Industries, US WorldMeds, Vista Research, WebMD, Windrose Consulting Group. Dr. Hauser reports research support from: AbbVie Inc., Acorda Therapeutics, AstraZeneca, Axovant Sciences, Biogen Inc., Cavion, Enterin Inc., Impax Laboratories, LLC., Intec Pharma Ltd, Jazz Pharmaceuticals, NeuroDerm Ltd., Lundbeck, Michael J Fox Foundation for Parkinson’s Research, F. Hoffman-La Roche, Dart NeuroScience LLC, Prexton Therapeutics, Revance Therapeutics Inc., Sunovion Pharmaceuticals Dr. Hauser reports grant support from: Parkinson’s Foundation

**Stuart Isaacson:** Honoraria for CME, consultant, research grants, and/or promotional speaker on behalf of: Abbvie, Acadia, Acorda, Adamas, Addex, Allergan, Amarantus, Axovant, Benevolent, Biogen, Britannia, Cerecor, Eli Lilly, Enterin, GE Healthcare, Global Kinetics, Impax, Intec Pharma, Ipsen, Jazz, Kyowa, Lundbeck, Michael J. Fox Foundation, Neurocrine, Neuroderm, Parkinson Study Group, Pharma2B, Roche, Sanofi, Sunovion, Teva, Theravance, UCB, US World Meds, Zambon

**Aaron Ellenbogen:** consulted for Adamas, Allergan, Amneal, Lundbeck, Neuroderm, US World

**Maria Angeles Rol and Juan Antonio Madrid:**

The authors declare no conflict of interest. JAM and MAR are founding partners of Kronohealth SL (a spin-off company participated also by the University of Murcia).

**Mark Lew:**

Advisor/consultant- Teva, US World Meds, UCB, Lundbeck, Abbvie, Adamas, Cynapsus, Revance, Acadia, Neurocrine, Acorda; Speaker- Teva, UCB, Lundbeck, Adamas, Acadia, Neurocrine; Research support: Parkinson’s Study Group, Michael J. Fox Foundation, Biotie, Neuroderm, Enterin Inc., Pharm2B, Cala Health; Fellowship Grants to Department of Neurology: Allergan, Medtronic

**Daniel Kremens:** Consultant/Speaker Bureau: UCB, Sunovion, Impax, Lundbeck , Acadia, US WorldMeds, Adamas, Abbvie, Merz, Allergan, Acorda, Kyowa, Neurocrine, GE Healthcare, St Jude Medical, Prexton. Research: Enterin, Revance

**Posner, Sutherland, Pagan, Li, Walter, Kumar, Vazquez, Oguh, Frucht, Maddux and Tse** have nothing to disclose.

**Author Roles**

(1) (A) conception and design of the study, (B) acquisition of data, (C) analysis and interpretation of data

(2) (A) drafting the article or (B) revising it critically for important intellectual content

(3) final approval of the version to be submitted

Robert A. Hauser 1A, 1B, 1C, 2A, 3

Dean Sutherland 1B, 2B, 3

Juan A. Madrid 1A, 1B, 1C, 2A, 3

Maria Angeles Rol 1A, 1B, 1C, 2A, 3

Steven Frucht 1B, 2B, 3

Stuart Isaacson 1B, 2B, 3

Fernando Pagan 1B, 2B, 3

Brian N. Maddux 1B, 2B, 3

George Li 1B, 2B, 3

Winona Tse 1B, 2B, 3

Benjamin L. Walter 1B, 2B, 3

Rajeev Kumar 1B, 2B, 3

Daniel Kremens 1B, 2B, 3

Mark F. Lew 1B, 2B, 3

Aaron Ellenbogen 1B, 2B, 3

Odinachi Oguh 1B, 2B, 3

Alberto Vasquez 1B, 2B, 3

William Kinney 1B, 2B, 3

Matt Lowery 1B, 2B, 3

Maria Resnick 1B, 2B, 3

Nicole Huff 1B, 2B, 3

Jerry Posner 1B, 2B, 3

Karla V. Ballman 1B, 2B, 3

Brian E. Harvey 1B, 2B, 3

Michael Camilleri 1A, 1B, 1C, 2A, 3

Michael Zasloff 1A, 1B, 1C, 2A, 3

Denise Barbut 1A, 1B, 1C, 2A, 3
